# Therapeutic Effects of Human Amniotic Fluid-Derived Stem Cells on Renal Interstitial Fibrosis in a Murine Model of Unilateral Ureteral Obstruction

**DOI:** 10.1371/journal.pone.0065042

**Published:** 2013-05-28

**Authors:** Dong Sun, Lin Bu, Caixia Liu, Zhongcheng Yin, Xudong Zhou, Xiaoju Li, Aiguo Xiao

**Affiliations:** 1 Department of Nephrology, Affiliated Hospital of Xuzhou Medical College, Xuzhou, P.R. China; 2 Department of Nephrology, Kuitun Hospital of Ili Kazakh Autonomous Prefecture, Kuitun, P.R. China; 3 Department of Internal Medicine and Diagnostics, Xuzhou Medical College, Xuzhou, P.R. China; Institut National de la Santé et de la Recherche Médicale, France

## Abstract

Interstitial fibrosis is regarded as the main pathway for the progression of chronic kidney disease (CKD) and is often associated with severe renal dysfunction. Stem cell-based therapies may provide alternative approaches for the treatment of CKD. Human amniotic fluid-derived stem cells (hAFSCs) are a novel stem cell population, which exhibit both embryonic and mesenchymal stem cell characteristics. Herein, the present study investigated whether the transplantation of hAFSCs into renal tissues could improve renal interstitial fibrosis in a murine model of unilateral ureteral obstruction (UUO). We showed that hAFSCs provided a protective effect and alleviated interstitial fibrosis as reflected by an increase in microvascular density; additionally, hAFSCs treatment beneficially modulated protein levels of vascular endothelial growth factor (VEGF), hypoxia inducible factor-1α (HIF-1α) and transforming growth factor-β1 (TGF-β1). Therefore, we hypothesize that hAFSCs could represent an alternative, readily available source of stem cells that can be applied for the treatment of renal interstitial fibrosis.

## Introduction

Chronic kidney disease (CKD), which affects individual worldwide [1], [2], is now recognized as a major public health problem. Interstitial fibrosis is regarded as the main pathway for CKD progression, which culminates in end-stage renal failure. Despite the great deal of effort invested in identifying therapies for CKD, the number of patients requiring dialysis and kidney replacement continues to rise [3]. Additionally, the current treatment modalities and donor kidney availability are insufficient, further increasing the demand for new available approaches to treat chronic nephropathy. Over the last decade, stem cells have become a promising therapeutic tool for the treatment of kidney diseases [4]–[7]. Several groups have demonstrated that bone marrow-derived mesenchymal stem cells (MSCs) contributed to regeneration following renal injury [8]–[11].

Stem cell-based therapies have gathered a substantial amount of interest due to their great potential for clinical applications. However, there are noticeable limitations in the use of adult stem cells (ASCs) and embryonic stem cells (ESCs) [12]. For example, ASCs, which exist in many adult tissues, cannot be effectively propagated; additionally, the use of ESCs is ethically controversial [13]. Although umbilical cord blood (UCB) has been demonstrated to be a promising source of fetal MSCs, the amount of MSCs in UCB is very low [14]. Recent reports have indicated that kidney stem cells have the ability to replace the damaged tubular epithelial cells [5], [15]. However, the regeneration capacity of the kidney is limited compared with other organs [16]. Consequently, it is imperative to search for a novel source of human stem cells that can be applied in regenerative treatments.

Amniotic fluid is commonly utilized in routine prenatal diagnostic tests and contains multiple cell types [17]. The stem cells derived from amniotic fluid have been induced to give rise to lineages from all three embryonic germ layers; further, over 90% of amniotic fluid-derived stem cells expressed the transcription factor Oct-4, a recognized marker of pluripotent human stem cells [18], [19]. Thus, human amniotic fluid-derived stem cells (hAFSCs) have become a promising stem cell source for cellular therapy because of their easy retrieval and the lack of ethical issues associated with their use [12], [20]. Previous studies have shown that hAFSCs may contribute to recovery following different types of kidney injuries [21], [22].

Hauser et al. [22] reported that the antiapoptotic effect of hAFSCs on tubular cells was significantly greater than MSCs. In our previous study [23], we transplanted endothelial progenitor cells (EPCs) into mice with unilateral ureteral obstruction (UUO); following transplantation, EPCs alleviated renal interstitial fibrosis. However, the isolation and culture of EPCs was very difficult. Is the culture of hAFSCs easier than EPCs? Is the therapeutic effect of hAFSCs on tubulointerstitial fibrosis better than that of EPCs?

In this study, hAFSCs were transplanted into nu/nu mice with unilateral ureteral obstruction (UUO) [24]. The abilities of transplanted hAFSCs to survive, accelerate the proliferation of tubular epithelial cells and prevent their apoptosis, and alleviate renal interstitial fibrosis were assessed.

## Results

### Phenotype, characterization and labeling of hAFSCs

**Figure 1:**
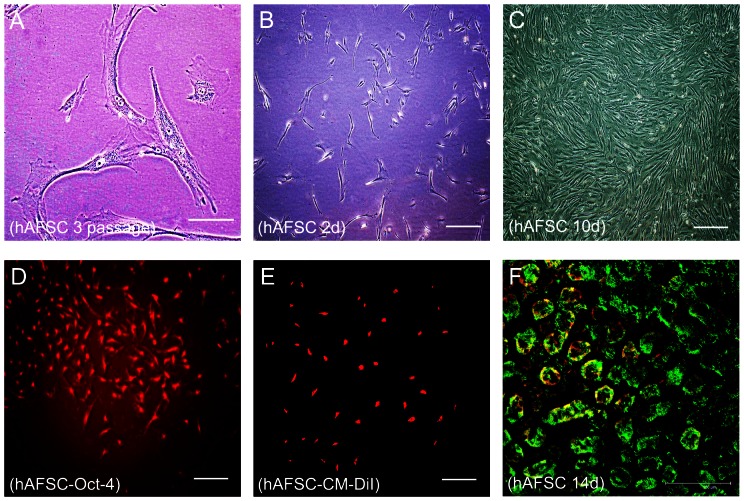
Culture, characterization and labeling of hAFSCs. (**A**) Morphology of hAFSCs under high magnification. After 3 passages in culture, the cells were spindle-shaped with relatively large nuclei; conjugate nuclei were also present (bar, 100 µm). (**B**) Morphology of hAFSCs after 2 days of culture. The cells exhibited a needle-like shaped appearance and grew with no visible directionality (bar, 50 µm). (**C**) Morphology of hAFSCs after 10 days of culture. Cells grow as a vortex and a crater (bar, 50 µm). (**D**) The third passage hAFSCs stained with Oct-4. Cells stained positive for Oct-4, a stem cell surface marker (bar, 50 µm). (**E**) CM-DiI-labeled hAFSCs. (**F**) Double immunofluorescent staining of a kidney section taken from an animal injected with hAFSCs at day 14 post-injection. Positive HLA-class I staining confirms the presence of hAFSCs at day 14 post-transplantation in the frozen renal tissue sections (bar, 50 µm).

Prior to injection, hAFSCs at the third passage were spindle-shaped with comparatively large nuclei; also, several hAFSCs exhibited conjugate nuclei (Figure 1A). At the second day of in vitro culture, hAFSCs exhibited a needle-like shape (Figure 1B). At the tenth day of in vitro culture, hAFSCs were confluent; however, they exhibited no visible directionality and grew as a vortex and a crater (Figure 1C). After the third passage, hAFSCs stained positive for Oct-4, a surface marker present on stem cells (Figure 1D). To examine intrarenal localization of hAFSCs after injection, cells were labeled with the cell surface marker CM-DiI (Figure 1E). Following transplantation, CM-DiI-positive cells were observed predominately in the tubular epithelial and in the interstitium; the human origin of the CM-DiI-positive cells was confirmed in kidney sections stained for human HLA class I at day 14 post-transplantation (Figure 1F). We found that hAFSCs can be propagated easily in vitro without the need of feeder layer and have a high proliferation potential compared with EPCs.

### Morphological Studies

**Figure 2:**
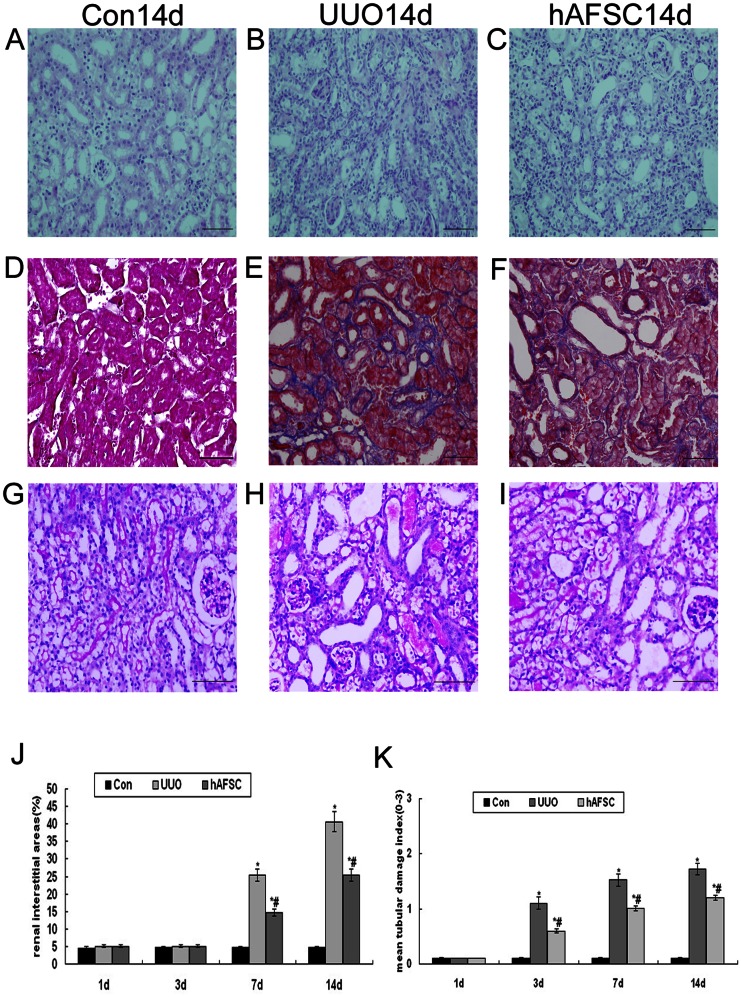
Morphological analysis of renal histology. HE and Masson's trichrome staining. (**A, D**) The normal control group at day 14 post-saline injection. No significant histological abnormalities were observed in the control group animals. (**B, E**) The UUO group at day 14 post-saline injection. Renal fibrosis was clearly visible in the UUO group animals at day 14. Increased numbers of collapsed vascellums and increased extracellular matrix production were observed. (**C, F**) The hAFSC group at day 14 post-hAFSCs injection. The number and the degree of destroyed tubules in the hAFSC group were less than the UUO group; further, renal fibrosis was reduced in the hAFSC group compared with the UUO group (bar, 50 µm). (**J**) Renal interstitium areas in each group. Fibrosis could be detected in the tubulointerstitium at day 7 and was more severe at day 14 in the UUO group. hAFSCs transplantation could significantly inhibit tubulointerstitial fibrosis (p<0.05). **G-I:** PAS staining. (**G**) The normal control group at day 14 post-saline injection. Normal morphology of a nu/nu mouse kidney was observed in the control group animals. The proximal and distal tubules as well as the glomeruli were intact. (**H**) The UUO group at day 14 post-saline injection. Marked disorganization of the structure of proximal and distal tubules with more cast formation and brush border disruption were observed. (**I**) The hAFSC group at day 14 post-hAFSCs injection. The tubular injury was observed, which showed less severe compared with the UUO group. (**K**) Results obtained with the tubular histology score. The loss of brush border, tubular dilation and apoptosis/necrosis of tubular cells were assessed. Values are presented as mean±SD. *p<0.05 vs. control group; #p<0.05 vs. UUO group.

To evaluate renal histology, paraffin-fixed biopsy samples were sectioned at a thickness of 5 µm and stained with hematoxylin-eosin and Masson's trichrome. As shown in Figure 2 A and D, there was no significant histological abnormalities in the control group. Some swelling of the renal tubular epithelial cells was observed at day 1 post-surgery in the UUO group. Although vacuole degeneration, disease expansion, inflammatory cell infiltration and the expansion of several tubules were detected at day 3 post-surgery in the UUO group, interstitial fibrosis was not obvious. At day 7 post-surgery, some tubular atrophy, blood vessel collapse, increased inflammatory infiltration, heightened interstitial fibrosis and increased extracellular matrix production were observed. Additionally, fibrosis was easily detected and more severe at day 14 post-surgery; further a majority of the tubules were destroyed at this time point (Figure 2 B, E). However, the number and the degree of tubular damage in the hAFSC group was less than that in the UUO group (Figure 2 C, F). As shown in Figure 2J, the interstitial area in the UUO group at day 7 and day 14 was significantly higher than that in the control group and in the hAFSC group, respectively. Moreover, the interstitial area at day 14 in the hAFSC group was markedly lower compared with the UUO group. Therefore, hAFSCs transplantation may have attenuated the development of renal interstitial fibrosis during UUO.

**Figure 3:**
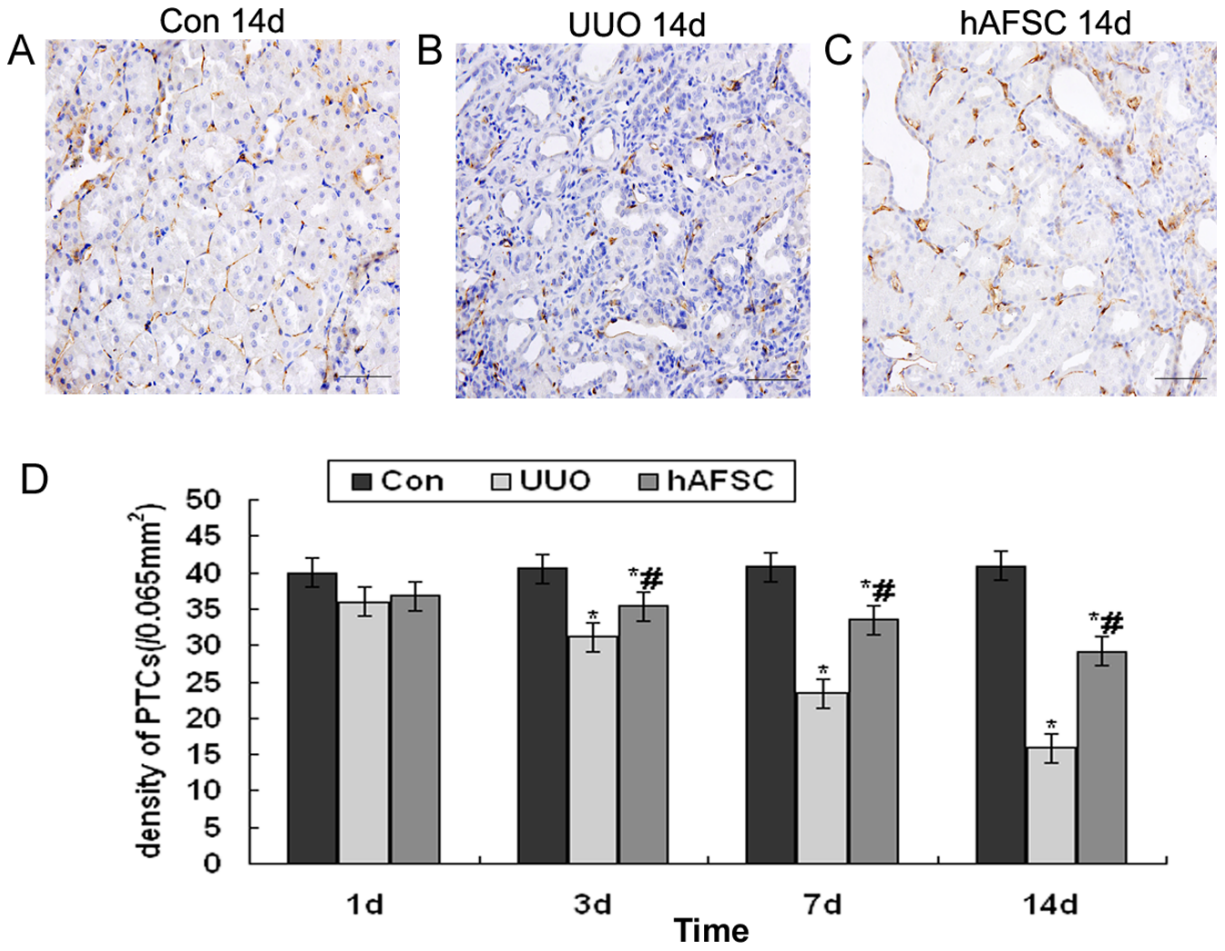
Changes in PTCs. (**A**) The normal control group at day 14 post-saline injection. The PTCs of the control group were easily identified and were uniform in size and shape; also, they were regularly arranged throughout the majority of the interstitium. (**B**) The UUO group at day 14 post-saline injection. The number of PTCs was significantly reduced in the UUO group, especially in the areas around the severely atrophic tubules. (**C**) The hAFSC group at day 14 post-hAFSCs injection. The number of PTCs in the hAFSC group was also reduced compared with the control group; however, the number of PTCs in the hAFSC group was greater than the UUO group (p<0.05) (bar, 50 µm). (**D**) PTC density in each group. The PTC density was significantly decreased beginning at day 3 in the UUO group. However, PTC density in the hAFSC group at day 3 was significantly increased compared with the UUO group (p<0.05). Values are presented as the mean±SD. *p<0.05 vs. control group; #p<0.05 vs. UUO group.

To assess the number and degree of the destroyed tubules, paraffin-fixed biopsy samples were sectioned at a thickness of 4 µm and stained with Periodic Acid Schiff (PAS). As shown in Figure 3G, the nu/nu mouse kidney showed normal morphology. The proximal and distal tubules as well as the glomeruli were intact. Following UUO, in obstructed kidneys, morphology of damaged mouse kidney at day 1 did not show significant differences compared to the control group. Tubular atrophy and dilation as well as tubular protein casts and interstitial fibrosis were observed from day 3. Marked disorganization of the structure of proximal and distal tubules with more cast formation and brush border disruption could be seen at day 14 post-surgery in the UUO group (Figure 2H, K). At day 1 post-surgery, there was no significant difference in tubules damage between the hAFSC and UUO groups. However, at day 3, day 7 and day 14 post-surgery, the hAFSC group showed less damage compared with the UUO group (Figure 2I, K). Taken together, the results demonstrated that hAFSCs transplantation may contribute to reduce the tubules damage in obstructive nephropathy in mice.

### Peritubular capillary (PTC) changes

To observe any changes in the microvasculature, immunostaining for CD34, which is expressed on vascular endothelial cells, was performed. As shown in Figure 3A, the PTCs in the control group samples were easily identified, uniform in size and shape and were regularly arranged throughout the majority of the interstitium. In contrast, the PTCs were damaged and the PTC density was significantly reduced beginning at day 3 post-surgery; further, the reduction in PTC density was more evident at day 14 post-surgery in the UUO group (Figure 3B). Moreover, the reduction in capillaries was particularly evident in areas of interstitial expansion and tubular atrophy. Although the number of PTCs in the hAFSC group was also reduced compared with the control group, PTC numbers in the hAFSC group were much higher than in the UUO group (Figure 3C). There was no significant difference in PTC density in the hAFSC group at day 1 compared with the UUO group. However, the PTC density in the hAFSC group was significantly increased at days 3, 7 and 14 post-surgery compared with the UUO group (Figure 3D). Taken together, these results demonstrated that hAFSCs transplantation may contribute to the angiogenesis observed in obstructive nephropathy in mice.

### Double Immunostaining and Western blot analysis of VEGF expression

**Figure 4:**
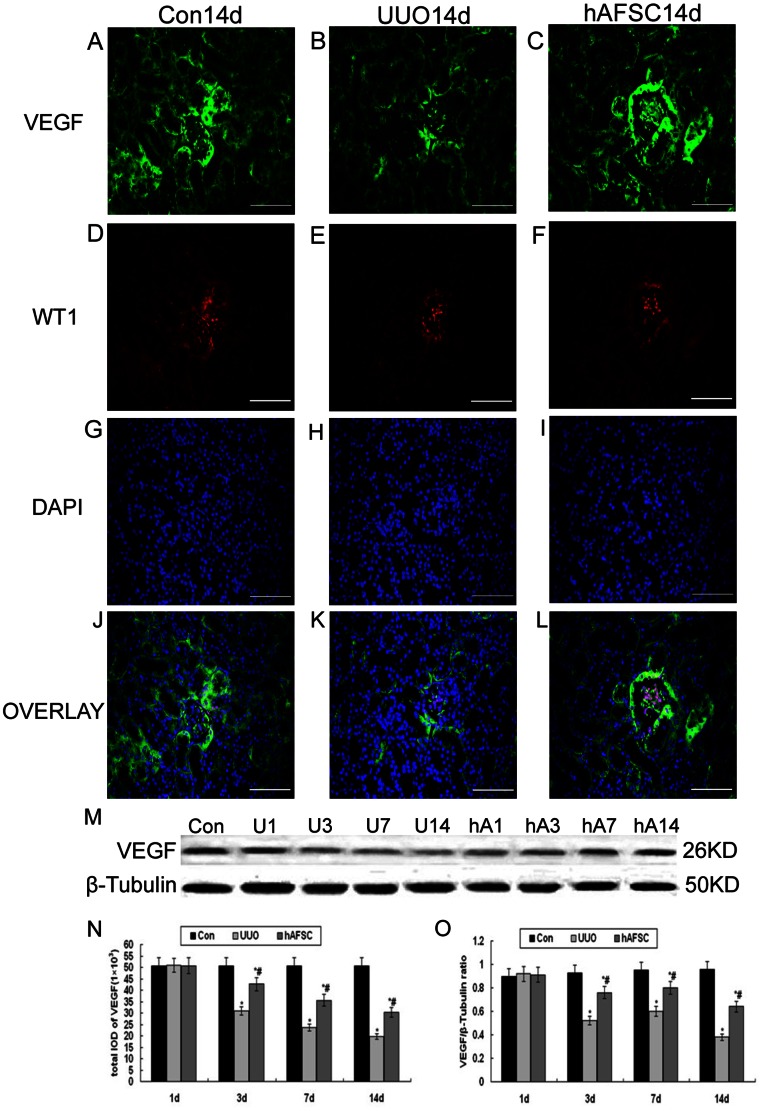
Double Immunostaining and Western Blot analysis of VEGF expression. (**A, J**) The normal control group at day 14 post-saline injection. VEGF was most prominently expressed in glomerular podocytes and tubular epithelial cells in the control group. (**B, K**) The UUO group at day 14 post-saline injection. VEGF expression gradually decreased over time post-surgery, particularly in the areas of tubular injury. (**C, L**) The hAFSC group at day 14 post-hAFSCs injection. VEGF expression was markedly enhanced in the hAFSC group (bar, 50 µm). (**N**) The total IOD of VEGF in each group. The total IOD of each group reflected the protein level. VEGF expression was notably enhanced in the hAFSC group compared with the UUO group (p<0.05). (**D, E, F**) WT-1 positive staining showed the podocytes (red). (**G, H, I**) DAPI positive staining showed the nucleuses (blue). (**M, O**) Western blot analysis of VEGF protein expression. VEGF protein expression showed similar trends with the double immunostaining findings. hAFSCs transplantation increased VEGF expression associated with UUO. Con, the control group at day 14; U1, U3, U7 and U14, the UUO group at days 1, 3, 7 and 14, respectively; hA1, hA3, hA7 and hA14, the hAFSC group at days 1, 3, 7 and 14, respectively. Values are presented as the mean±SD. *p<0.05 vs. control group; #p<0.05 vs. UUO group.

Double immunostaining for both VEGF and Wilms tumor1 (WT-1) revealed that the control group had the most prominent VEGF expression in glomerular podocytes and tubular epithelial cells (Figure 4A, J), and that the expression of VEGF increased at day 1 in the UUO group and hAFSC group, which did not show significant differences compared with the control group. However, VEGF expression gradually decreased in the days post-surgery, particularly in the areas of tubular injury; the reduction in VEGF expression was more noticeable in the UUO group (Figure 4B, K) than that in the hAFSC group (Figure 4C, L). Although the levels of VEGF were significantly reduced in both the UUO and hAFSC groups at day 3, 7 and 14 compared with the control group, VEGF expression was higher in the hAFSC group compared with the UUO group (Figure 4N). The results of the western blot analysis (Figure 4M, O) were similar to the double immunostaining results. These data suggest that hAFSCs transplantation promoted VEGF expression.

### Immunohistochemical staining and Western blot analysis of HIF-1α expression

**Figure 5:**
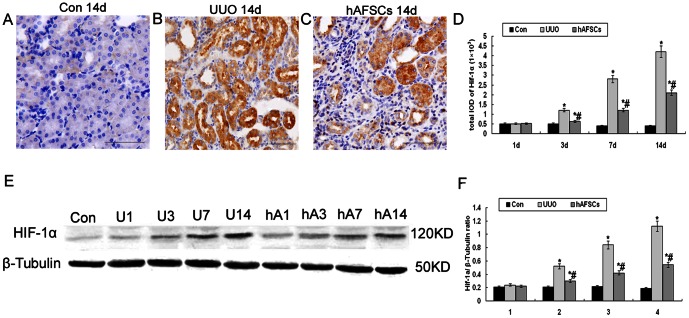
Immunohistochemical staining and Western blot analysis of HIF-1α expression. (**A**) The normal control group at day 14 post-saline injection. HIF-1α staining was faint in the cortical nuclei in the control group at day 14 post-saline injection; nuclear staining was not observed in the medulla. (**B**) The UUO group at day 14 post-saline injection. HIF-1α expression was most apparent in areas of interstitial fibrosis and tubular dilation in the UUO group at day 14. (**C**) The hAFSC group at day 14 post-hAFSCs injection. HIF-1α expression was suppressed in renal tissues after hAFSCs transplantation (bar, 50 µm). (**D**) The total IOD of HIF-1α in each group. The total IOD in each group reflected the levels of HIF-1α protein. HIF-1α expression was decreased in tubules after hAFSCs transplantation beginning at day 3 compared with the UUO group (p<0.05). (**E, F**) Western blot analysis of HIF-1α protein expression. HIF-1α protein expression was significantly reduced in the hAFSC group at day 3 post-surgery compared with the UUO group and remained low at days 7 and 14 post-surgery; these results are consistent with the immuno-histochemistry findings. hAFSCs transplantation alleviated HIF-1α expression associated with UUO. Con, the normal control group at day 14; U1, U3, U7 and U14, the UUO group at days 1, 3, 7 and 14, respectively; hA1, hA3, hA7 and hA14, the hAFSC group at days 1, 3, 7 and 14, respectively. Values are presented as the mean±SD. *p<0.05 vs. control group; #p<0.05 vs. UUO group.

As shown in Figure 5A, HIF-1α staining was faint in the cortical nuclei in the control group. There was no nuclear staining in glomeruli and medulla or papilla. HIF-1α expression was similar among the three groups at day 1 post-surgery. In the UUO group, HIF-1α expression was increased in tubular epithelial cells and fibrotic areas at day 3 compared with the control and hAFSC groups; this increase in HIF-1α expression in the UUO group was even more pronounced at days 7 and 14 post-surgery (Figure 5B, D). However, HIF-1α expression was apparently decreased in tubules after hAFSCs transplantation beginning at day 3 post-surgery, especially in the areas of atrophy and fibrosis (Figure 5C). Western blot analysis results showed that the HIF-1α protein levels were decreased in the hAFSC group at day 3 post-surgery compared with the UUO group and remained low at days 7 and 14 post-surgery (Figure 5E, F). These results were consistent with the immunohistochemistry findings and clearly indicated that hAFSCs transplantation alleviated the hypoxic conditions associated with UUO.

### Immunohistochemical staining and Western blot analysis of TGF-β1 expression

**Figure 6:**
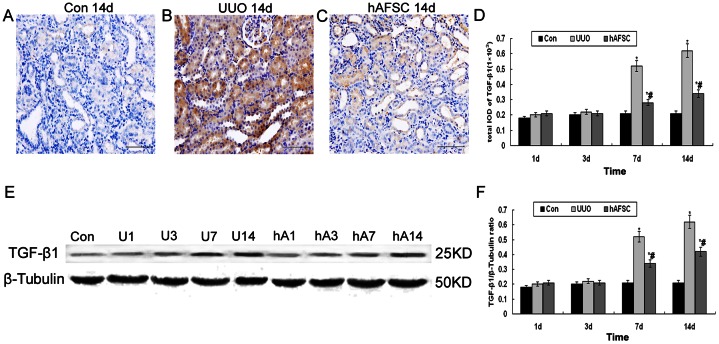
Immunohistochemical staining and Western blot analysis of TGF-β1 expression. (**A**) The normal control group at day 14 post-saline injection. Faint TGF-β1 staining was observed in renal tissues from normal control group mice at day 14 post-saline injection. (**B**) The UUO group at day 14 post-saline injection. TGF-β1 was markedly upregulated in the interstitial fibrotic areas and tubular epithelial cells in the UUO group at day 14. (**C**) The hAFSC group at day 14 post-hAFSCs injection. TGF-β1 expression was suppressed following hAFSCs transplantation (bar, 50 µm). (**D**) The total IOD for TGF-β1 in each group. The total IOD in each group reflected the level of TGF-β1 protein expression. TGF-β1 protein levels in the UUO group began to increase at day 7 and remained elevated at day 14 compared with the hAFSC group (p<0.05). (**E, F**) Western blot analysis of TGF-β1 protein expression. Western blot analysis showed that TGF-β1 protein levels began to increase at day 7 and remained elevated at day 14; these results were consistent with the immunohistochemistry findings. hAFSCs transplantation reduced TGF-β1 expression associated with UUO. Con, the normal control group at day 14; U1, U3, U7 and U14, the UUO group at days 1, 3, 7 and 14, respectively; hA1, hA3, hA7 and hA14, the hAFSC group at days 1, 3, 7 and 14, respectively. Values are presented as the mean±SD. *p<0.05 vs. control group; #p<0.05 vs. UUO group.

In the control group, faint TGF-β1 staining was observed in the renal tissue (Figure 6A). However, markedly increased TGF-β1 immunostaining was clearly detected in tubular epithelial cells, interstitial cells, interstitial fibrotic regions and areas of glomerulosclerosis at days 7 and 14 post-surgery in the UUO group (Figure 6B). TGF-β1 expression began to increase at day 7 post-surgery and reached a high level at day 14 post-surgery in the UUO group. Although there was no significant difference in TGF-β1 expression at days 1 and 3 post-surgery between the UUO and hAFSC groups, TGF-β1 protein expression was markedly reduced at days 7 and 14 post-surgery in the hAFSC group compared with the UUO group (Figure 6C). Western blot analysis revealed that TGF-β1 protein levels began to increase at day 7 and remained at a high level at day 14 in the UUO group (Figure 6E, F), and the results were consistent with the immunohistochemistry findings. These data clearly indicated that hAFSCs transplantation might reduce TGF-β1 expression in a murine model of UUO.

### Immunohistochemical staining and Western blot analysis of E-cadherin expression

**Figure 7:**
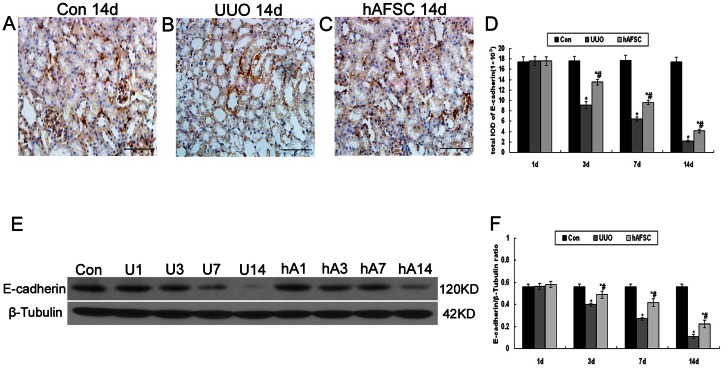
Immunohistochemical staining and Western blot analysis of E-cadherin expression. (**A**) Normal control group at 14 days after saline solution injection: Positive staining of E-cadherin was seen in the tubuloepithelial cells. (**B**) UUO group at 14 days after saline solution injection: Rare expression of E-cadherin was seen at day 14 after surgery, and most renal tubules were destroyed and renal interstitial fibrosis was obvious. (**C**) hAFSC group at 14 days after hAFSCs injection: The expression of E-cadherin increased after hAFSCs transplantation. (bar, 50 µm). (**D**) The total IOD of E-cadherin in each group: The total IOD in each group reflected the level of the E-cadherin. There was no significant difference in the expression of E-cadherin among the UUO, hAFSC, and control groups 1 day after surgery. The expression of E-cadherin began to reduce from 3 days after surgery. Rare expression of E-cadherin was seen at 14 days after surgery in the UUO group. However, the expression of E-cadherin significantly increased in the hAFSC group compared to the UUO group(p<0.05). (**E, F**) Western blot analysis for E-cadherin protein expression. E-cadherin protein expression showed the similar trends as the immunohistochemistry findings. hAFSCs transplantation increased E-cadherin expression compared with UUO at day 3, day 7 and day 14. Con, the control group at day 14; U1, U3, U7 and U14, the UUO group at days 1, 3, 7 and 14, respectively; hA1, hA3, hA7 and hA14, the hAFSC group at days 1, 3, 7 and 14, respectively. Values are presented as mean±SD. *p<0.05 vs. control group; #p<0.05 vs. UUO group.

The results showed that E-cadherin expressed predominantly in cytoplasmic membrane. In the control group, positive staining of E-cadherin was seen in the tubuloepithelial cells(Figure 7A). There was no significant difference in the expression of E-cadherin among the UUO group, hAFSC group, and control group at day 1 after surgery. The expression of E-cadherin began to reduce from day 3 after surgery. Along with the obstruction progressed, renal tubules were destroyed more severely, which resulted in a low expression of E-cadherin (Figure 7D). Moreover, few E-cadherin positive cells were seen 14 days after surgery, most renal tubules were destroyed, and renal interstitial fibrosis were obvious (Figure 7B). Although the expression of E-cadherin in tubuloepithelial cells in the hAFSC group significantly reduced from the day 3 after surgery compared with the control group, the expression of E-cadherin in the hAFSC group was significant higher than that in the UUO group (Figure 7C, D). Western blot analysis of E-cadherin protein expression showed that E-cadherin expression began to decrease from day 3 after surgery, which was consistent with the immunohistochemistry findings (Figure 7E, F). These data clearly indicated that hAFSCs transplantation might increased E-cadherin expression in a murine model of UUO.

### Immunohistochemical staining and Western blot analysis of Collagen-I expression

**Figure 8:**
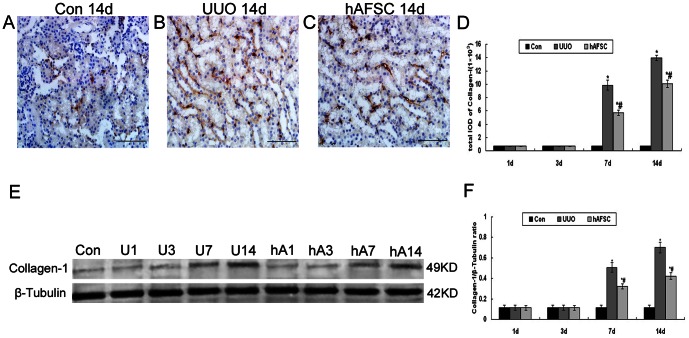
Immunohistochemical staining and Western blot analysis of Collagen I expression. (**A**) Normal control group at 14 days after saline solution injection: Light staining of Collagen I was seen in renal interstitium. (**B**) UUO group at 14 days after saline solution injection: The expression of Collagen I significant increased at day 14 after surgery in the UUO group, and the renal interstitial fibrosis were more severe. (**C**) hAFSC group at 14 days after hAFSCs injection: The expression of Collagen I reduced after hAFSC transplantation. (bar, 50 µm). (**D**) The total IOD of Collagen I in each group: The total IOD in each group reflected the level of the Collagen I. There was no significant difference in the expression of Collagen I among the UUO, hAFSC, and control groups at 1 day and 3 days after surgery. The expression of Collagen I significantly increased at 7 and 14 days after surgery in the UUO group and hAFSC group compared to the control group, respectively. However, hAFSC transplantation significantly resulted in a reduction of the expression of Collagen I compared to the UUO group. (**E, F**) Western blot analysis of Collagen I protein expression. Western blot analysis showed that Collagen I protein levels began to increase at day 7, and remained elevated at day 14; these results were consistent with the immunohistochemistry findings. hAFSCs transplantation reduced Collagen I expression associated with UUO. Con, the normal control group at day 14; U1, U3, U7 and U14, the UUO group at days 1, 3, 7 and 14, respectively; hA1, hA3, hA7 and hA14, the hAFSC group at days 1, 3, 7 and 14, respectively. Values are presented as the mean±SD. *p<0.05 vs. control group; #p<0.05 vs. UUO group. Values are presented as the mean±SEM. *p<0.05 vs. control group; #p<0.05 vs. UUO group.

In the control group, light staining of Collagen I was seen in renal interstitium (Figure 8A). There was no significant difference in the expression of Collagen I among the UUO group, hAFSC group, and control group at day 1 and day 3 after surgery. The expression of Collagen I significantly increased from day 7 after surgery in the UUO group and hAFSC group, which concentrated in the fibrotic areas. Along with the obstruction progressed, renal interstitial fibrosis were more severe, and the expression of Collagen I significantly increased at day 14 after surgery in the UUO group and hAFSC group compared with the control group (Figure 8B-D). However, hAFSCs transplantation significantly reduced the expression of Collagen I compared with the UUO group (Figure 8D). Western blot analysis of Collagen I protein expression showed that Collagen I expression began to increase at day 7 and remained elevated at day 14 after surgery, which were consistent with the immunohistochemistry findings (Figure 8E, F). These data clearly indicated that hAFSCs transplantation might reduced Collagen I expression in a murine model of UUO

### Immunohistochemical staining and Western blot analysis of monocyte chemoattractant protein-1 (MCP-1) expression

**Figure 9:**
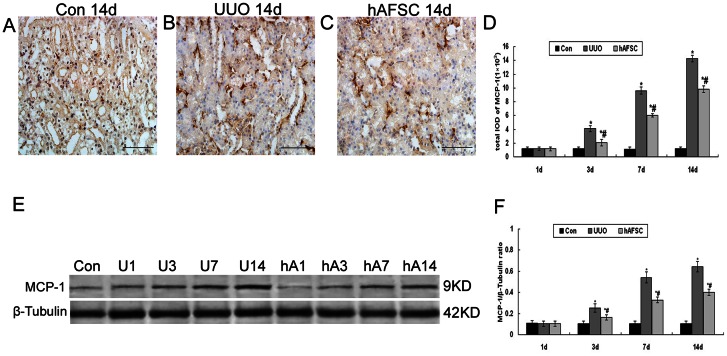
Immunohistochemical staining and Western blot analysis of MCP-1 expression. (**A**) Normal control group at 14 days after saline solution injection: Light staining of MCP-1 was seen in the renal tissue and a few inflammatory cells and fibrosis were seen in tubulointerstitial. (**B**) UUO group at 14 days after saline solution injection: The expression of MCP-1 significant increased in the UUO group at 14 days after surgery. (**C**) hAFSC group at 14 days after hAFSCs injection: The expression of MCP-1 reduced after hAFSCs transplantation. (bar, 50 µm). (**D**) The total IOD of MCP-1 in each group: The total IOD in each group reflected the level of the MCP-1 protein. The expression of MCP-1 in the UUO group and hAFSC group at day 1 after surgery was not obvious. There was no significant difference in the expression of MCP-1 among the UUO, hAFSC, and control groups at 1 day after surgery. However, the expression of MCP-1 significantly increased in the UUO group and hAFSC group at 3, 7 and 14 days after surgery compared to the control group, respectively. hAFSCs transplantation significantly reduced the tubuloepithelial cell expression of MCP-1 compared to the UUO group. (**E, F**) Western blot analysis of MCP-1 protein expression. Western blot analysis showed that MCP-1 protein levels began to increase at day 3, and remained elevated at day 14; These results were consistent with the immunohistochemistry findings. hAFSCs transplantation reduced MCP-1 expression associated with UUO(*P*<0.05). Con, the normal control group at day 14; U1, U3, U7 and U14, the UUO group at days 1, 3, 7 and 14, respectively; hA1, hA3, hA7 and hA14, the hAFSC group at days 1, 3, 7 and 14, respectively. Values are presented as the mean±SD. *p<0.05 vs. control group; #p<0.05 vs. UUO group. Values are presented as the mean±SEM. *p<0.05 vs. control group; #p<0.05 vs. UUO group. Values are presented as the mean±SEM. *p<0.05 vs. control group; #p<0.05 vs. UUO group.

In the control group, light staining of MCP-1 was seen in the renal tissue and a few inflammatory cells and fibrosis were seen in tubulointerstitium (Figure 9A). The expression of MCP-1 in the UUO group and hAFSC group at day 1 after surgery was not obvious. There was no significant difference in the expression of MCP-1 among these two groups and the control group at day 1 after surgery. In contrast, we found that the expression of MCP-1 significantly increased in the UUO group and hAFSC group at day 3, 7 and 14 after surgery compared with the control group (Figure 9D). However, hAFSCs transplantation significantly reduced the expression of MCP-1 in tubuloepithelial cell compared with the UUO group (Figure 9B-D). Western blot analysis of MCP-1 protein expression showed that MCP-1 expression began to increase at day 3 and remained elevated at day 14 after surgery, which were consistent with the immunohistochemistry findings (Figure 9E, F). These data clearly indicated that hAFSCs transplantation might reduced MCP-1 expression in a murine model of UUO.

### Proliferation (PCNA and Ki67 Staining) and Apoptosis (TUNEL Staining)

**Figure 10:**
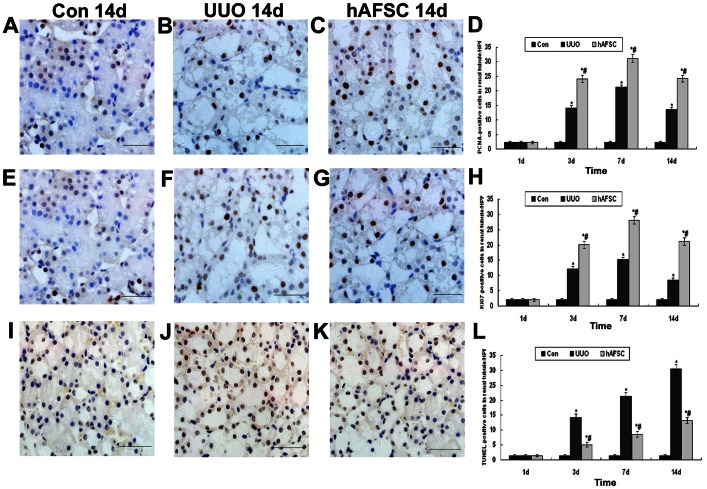
Proliferation and apoptosis evaluation. (**A, E**) Normal control group at 14 days after saline solution injection: The proliferation activity in tubular cells showed no obvious increase in the control group. (**B, F**) UUO group at 14 days after saline solution injection: The proliferation activity in tubular cells showed significant increased in the UUO group at 14 days after surgery. (**C, G**) hAFSC group at 14 days after hAFSCs injection: Significantly more proliferation in tubular cells could be seen in the hAFSC group. (bar, 50 µm). (**D, H**) PCNA-positive cells and Ki67-positive cells in renal tubules in each group. The proliferation activity among the three experimental groups showed no significant difference at day 1 post-surgery. Animals that were injected with a saline solution and animals that received hAFSCs after UUO demonstrated a significant increase in cell proliferation beginning at day 3 post-surgery. The hAFSC group had significantly more proliferation than the UUO group. Although the proliferation activity of the hAFSC group at day 14 post-surgery showed an obvious decrease than that at day 7 post-surgery, it still showed a significant increase than the UUO group at day 14. (**I**) Normal control group at 14 days after saline solution injection: The apoptosis activity in tubular cells showed no obvious increase in the control group. (**J**) UUO group at 14 days after saline solution injection: The apoptotic cells showed significant increased in UUO group at 14 days after surgery. (**K**) hAFSC group at 14 days after hAFSCs injection: The apoptotic cells were significantly decreased in animals in the hAFSC group (bar, 50 µm). (**L**) TUNEL-positive cells in renal tubules in each group. The apoptosis activity among the three experimental groups showed no significant difference at day 1 post-surgery. Animals that were injected with a saline solution and animals that received hAFSCs after UUO demonstrated a significant increase in cell apoptosis beginning at day 3 post-surgery. Although the apoptosis activity of the hAFSC group at day 14 post-surgery showed an obvious increase than that at day 7 post-surgery, it still showed a significant decrease than the UUO group at day 14. Values are presented as the mean±SEM. *p<0.05 vs. control group; #p<0.05 vs. UUO group.

The proliferation and apoptosis of tubular cells were compared among the experimental groups at days 1, 3, 7 and 14 post-surgery. There was no obvious increase in the proliferative activity of tubular cells in the control mice without UUO (Figure 10A, E). The proliferation activity among the three experimental groups showed no significant difference at day 1 post-surgery. In contrast, animals that were injected with a saline solution and animals that received hAFSCs after UUO demonstrated a significant increase in cell proliferation beginning at day 3 post-surgery (Figure 10D, H). The hAFSC group had significantly more proliferation than the UUO group (Figure 10B, C, F, G). Although the proliferation activity of the hAFSC group at day 14 post-surgery showed an obvious decrease than that at day 7 post-surgery, it still showed a significant increase than the UUO group at day 14 (Figure 10D, H).

As shown in Figure 10 I-L, apoptosis was significantly increased in animals in the UUO and hAFSC groups compared with the control group. Moreover, with the ligation last, the renal damage was more severe and many apoptotic cells were observed beginning at day 3 in the UUO group. However, the number of apoptotic cells was significantly decreased in animals in the hAFSC group compared with the UUO group.

## Discussion

CKD is increasingly being recognized as a major public health problem. Interstitial fibrosis is regarded as the main pathway for CKD progression, which culminates in end-stage renal failure. Convincing evidence also exists that CKD is a high-risk factor for cardiovascular disease [25] and stroke [26]. Therefore, it has been imperative to explore novel feasible methods for CKD treatment. Over the last several decades, cell-based therapies have been applied to repair kidney damage. A number of studies have shown that transplantation of mesenchymal stem cells (MSCs) into injured kidneys contributed to renal repair [9], [22], [27]–[31]. Nevertheless, the use of these cells in humans involves both ethical and political hurdles [32].

hAFSCs have advantages over other stem cells and have become a promising stem cell source for use in clinical applications [33]. In this study, we evaluated hAFSCs as a novel stem cell population for use in cell-based therapeutics.

PTC injury is known to be associated with a reduced blood supply to the renal tubules [34]. The number of PTCs detected using CD34 immunostaining in our study showed that the PTCs were damaged in the UUO group; PTC density was significantly reduced beginning at day 3 post-surgery, with reductions in PTC density becoming more evident at day 14 post-surgery in the UUO group. However, PTC density in the hAFSC group was higher compared with the UUO group at days 7 and 14 post-surgery. Therefore, we hypothesized that hAFSCs transplantation had the ability to repair renal blood vessels and stimulate an increase in PTC density in UUO mice.

Recent studies have suggested that VEGF has the ability to enhance glomerular capillary repair and prevent the progression of kidney disease [35], [36]. VEGF expression levels were gradually reduced as the renal damage progressed in the UUO and hAFSC groups; however, the VEGF expression in the UUO group was less than the hAFSC group. Interestingly, VEGF expression appeared to be increased at day 1 post-surgery. This result indicates that VEGF expression may be a compensatory response to obstructive renal disease. hAFSCs have been demonstrated to exhibit a protective effect in kidney injury models. Several studies have suggested that MSCs have the ability to express growth factors such as VEGF, HGF and IGF-1 [37], [38]. Consequently, the detection of reduced VEGF levels in UUO could be the result of multiple factors in addition to hypoxia and HIF-1 expression [29]. Our results show that hAFSCs transplantation into kidneys may contribute to angiogenesis. Nevertheless, the concrete mechanisms involved in this process remain to be investigated in further studies.

Hypoxia is recognized as an essential factor in the dysregulation of angiogenesis. PTC injury may lead to chronic ischemia and hypoxia [39], thus accelerating the process of fibrosis [40], [41]. HIF-1α is a sensitive indicator of hypoxia that remains stable in low oxygen conditions [42]. HIF-1α expression was extremely low in the control group. In contrast, its expression gradually increased in the UUO group over time. HIF-1α levels were markedly decreased in the hAFSC group compared with the UUO group, indicating that hAFSCs transplantation may improve hypoxic tubulointerstitial conditions in UUO.

TGF-β1 has been generally recognized as the most important factor leading to fibrosis, and it plays a significant role in the progression of renal interstitial fibrosis [43]. It has been suggested that once enough renal mass is lost, the angiotensin-aldosterone system (RAAS) is activated, which subsequently induces TGF-β1 and extracellular matrix production [16], further accelerating renal mass loss. In our present study, TGF-β1 expression was significantly increased in the UUO group compared with the control group. However, TGF-β1 levels in the hAFSC group were lower than in the UUO group.

To inject hAFSCs into mice and avoid rejection, we utilized nu/nu mice in this study. Nu/nu mice are immunodeficient as they lack activated T lymphocytes; however, they have normal B lymphocytes and NK cells, which provides the mice with some ability to mount an immune response when stimulated [44]. In our study, hAFSCs were labeled with the cell surface marker CM-DiI prior to being transplanted on the same day of UUO induction. The human origin of the CM-DiI-positive cells was confirmed in kidney sections at day 14 using human HLA class I antigen staining. CM-DiI-positive cells persisted following transfer and were detected at day 14 post-transplantation; they were predominately observed in the tubular epithelial and the interstitium, indicating that hAFSCs could traffic into the obstructed kidney [22].

To investigate the effect of hAFSCs transplantation on the interstitial inflammation, we examined the levels of MCP-1, one of the most important indicators involving in the inflammatory process within the renal tubulointerstitial tissue [45]. In our study, the expression of MCP-1 significantly reduced in the hAFSC group compared with the UUO group, which indicated that hAFSCs transplantation attenuated the interstitial inflammation.

Cadherins are considered to be crucial for tissue morphogenesis in various contexts [46]. In our study, we focused on E-cadherin, the predominant family member of the classic cadherins in tubuloepithelial homeostasis. The expression of E-cadherin significantly increased in the hAFSC group compared with the UUO group, which could be interpreted as that hAFSCs transplantation may prevent the renal tissue morphogenesis from further damage.

Collagen accumulation in fibrosis has been considered to be a marker for the balance between synthesis and degradation [47]. Collagen I is produced by tubular interstitial cells and leads to renal fibrosis. Our results showed that the expression of collagen I in the UUO group increased, especially at day 14 after UUO. Interestingly, the expression of collagen in the hAFSC group was significant reduced compared with the UUO group. The data demonstrated that hAFSCs transplantation could decrease the collagen accumulation in fibrotic areas, and then alleviate renal interstitial fibrosis.

In our study, we examined the proliferation and apoptosis of tubular cells within the three experimental groups to evaluate the therapeutic effect of hAFSCs injection. The results demonstrated that hAFSCs injection might accelerate the proliferation of tubular epithelial cells that were partially damaged and possibly prevent their apoptosis. We hypothesize that the mechanisms involved in the tubular epithelial cell proliferation and apoptosis were paracrine in nature; however, these mechanisms need to be studied in the future.

The mechanisms involved in the repair of kidney injury vary according to the type of damage. It has been reported that injection of hAFSCs into injured kidneys resulted in an increase in anti-inflammatory cytokine production, which could help ameliorate the acute phase of injury [21]. Resident epithelial cell proliferation has also been considered to be the main repair mechanism in a model of ischemic-induced tubular injury [48]. Additionally, the paracrine action was confirmed to play a critical role in recovery from experimental acute kidney injury [49]. However, the specific mechanisms underlying kidney repair remain to be further investigated.

In our previous study [23], we transplanted EPCs into mice with unilateral ureteral obstruction and alleviated renal interstitial fibrosis. In our present study, we demonstrated the transplantation of hAFSCs into nu/nu mice had a higher ability to repair renal blood vessels compared with EPCs transplantation; further, the degree of renal interstitial fibrosis was less in mice treated with hAFSCs compared with mice treated with EPCs.

In conclusion, our present study indicated that transplanted hAFSCs could alleviate the progression of renal fibrosis by elevating VEGF protein levels, increasing PTC density and decreasing the expression of HIF-1α and TGF-β1. Moreover, hAFSCs transplantation could partially accelerate the proliferation of tubular epithelial cells and prevent their apoptosis.

Therefore, we conclude that hAFSCs may provide a promising and valuable cell-based therapeutic tool for the treatment of kidney disease.

## Materials and Methods

### hAFSCs Isolation, Culture and Labeling

Seeds of hAFSCs were obtained from the Chinese Academy of Sciences, Beijing. After being revived from liquid nitrogen storage, cells were centrifuged and plated in 5 ml of a-modified minimum essential medium (a-MEM) (Gibco-BRL) supplemented with 20% fetal bovine serum (FBS) (Gibco/BRL), 4 ng/ml basic fibroblast growth factor (bFGF) (R&D systems, Minneapolis, MN), 100 µg/ml glutamine (Sigma-Aldrich) and 1% antibiotics (pen-strep) (Gibco/BRL) in 25 cm^2^ T-flasks and incubated at 37°C in a humidified atmosphere with 5% CO_2_; the culture medium was changed every 2 or 3 days.

Prior to injection, the fifth passage of hAFSCs were trypsinized in 0.05 M trypsin/EDTA (Gibco/BRL) solution and centrifuged at 1200 rpm for 5 min. To enable visualization after injection, cells were labeled with the cell surface marker CM-DiI (Molecular Probe) according to the manufacturer's instructions. Briefly, the cells were incubated with a working solution of 1 mg/ml of CM-DiI for 10 min at 37°C followed by incubation at 4°C for 15 min; cells were then washed three times with phosphate-buffered saline (PBS) (Gibco/BRL). Next, the cells were injected into UUO mice. To examine the intrarenal localization of the cells on days 1, 3, 7 and post-injection, mice were sacrificed, and renal samples were embedded in OCT and snap-frozen in liquid nitrogen. To confirm the human origin of the hAFSCs, tissue sections (5 µm in thickness) were fixed in acetone for 10 min and double-stained using monoclonal anti-cytokeratin antibodies (Sigma Aldrich) and polyclonal anti-HLA class I antibodies (Santa Cruz Biotechnology). All sections were then observed using a fluorescence microscope.

### Immunofluorescent staining of hAFSCs

To verify that the provided cells were hAFSCs, cell markers were characterized using fluorescence microscope detection. Several groups [18], [19] have reported that Oct-4 is a marker of pluripotent stem cells. For immunofluorescence analyses of cellular Oct-4 expression, cultured hAFSCs at the third passage were fixed in 4% paraformaldehyde (Germany) after they had grown to 70% confluence. Cells were permeabilized with 0.2% Tritonx-100 and then stained overnight with a primary antibody against Oct-4 (1∶400) (Zhong Shan, Bei Jing). The cells were then washed three times with PBS-Tween [PBS with 0.1% Tween-20 (UBS, OH)] before being stained with a 1∶200 dilution of goat anti-rabbit IgG secondary antibody (Chenicon, MA). Staining patterns were then visualized using an inverted fluorescent microscope.

### Animal model and hAFSCs injection

Nu/nu mice (body weight: 18–22 g, age: 6–8 weeks) were purchased from the Laboratory Animal Centre of Xuzhou Medical College (Jiangsu, China). The Institutional Animal Care of Xuzhou Medical College approved all surgical interventions and post-operative animal care. The approved protocol number is SYXK (SU) 2005-0018. The approval date for this experiment was Sep 18^th^, 2010. The experimental animals were randomized into the following three groups: the normal control group (saline solution), the UUO group (UUO + saline solution) and the hAFSC group (UUO + hAFSCs). Each group contained 32 animals.

Renal interstitial fibrosis was induced in female nu/nu mice by ligating half of the ureter according to a method described previously [24]. Briefly, the mice were carefully anesthetized using chloral hydrate (10.0%); after satisfactory anesthesia was achieved, the mice were prepared for surgery. A 1 cm ventral incision was made, the left ureter and kidney were dissociated, and the lower third of the ureter was ligated with a suture (4/0). The incision was then closed using polypropylene sutures, and the mice were allowed to recover from anesthesia. At the same time, animals in the hAFSC group were intravenously injected through the tail vein with 3.5×10^5^ CM-DiI-labeled hAFSCs in 150 µl of PBS. As a control, animals in the UUO group were injected with a saline solution. Animals in the control group underwent no surgical manipulation and received saline solution only.

At days 1, 3, 7 and 14 post-surgery, mice from the three groups were sacrificed. The left kidneys were extracted and washed in cold saline solution. One portion of each kidney was fixed in 10% formaldehyde for pathological examinations and immunohistochemical detection, while another portion was stored in liquid nitrogen or at −80°C for later western blot analysis.

### Histological examinations

Specimens from each group were taken at days 1, 3, 7 and 14 post-surgery. The specimens used for pathological examinations were fixed in 10% formaldehyde for 24 hours at room temperature, dehydrated using a gradual alcohol series, embedded in paraffin, and sectioned into 4 µm thick sections. The sections were deparaffinized and then incubated for 15 min in HistoChoice. Subsequently the sections were sequentially incubated for 5 min in 100% alcohol, 95% alcohol, 85% alcohol, 75% alcohol and water. The sections were then stained with hematoxylin-eosin and Masson's trichrome [50]. The areas of the fibrotic kidney lesions were determined using Masson's trichrome staining to visualize the collagen fibers, which were stained dark blue. Under higher-power magnification (×40), ten discontinuous visual fields of the outer cortex of the kidney on each section were selected randomly. The fibrotic areas stained in dark blue were identified in digital images, and the percentage of the fibrotic areas relative to the whole area of the field was calculated; glomeruli and large vessels were not included in the analysis.

To assess the number and degree of destroyed tubules, specimens from each group were fixed in 4% formaldehyde in PBS for 8 hours at 4°C, dehydrated through a gradual alcohol series, embedded in paraffin, and sectioned into 4 µm thick sections. The sections were deparaffinized and then incubated for 15 min in HistoChoice, then 5 min in 100% alcohol, 95% alcohol, 85% alcohol, 75% alcohol and then water. The sections were then stained with PAS (Sigma-Aldrich) for 5 min at room temperature and counterstained with hematoxylin. The tubular injury was scored on a scale of 0–3∶ 0 = normal histology; 1 = mild dilatation; 2 = flattened epithelial cells and loss of brush border; 3 = denudation of basement membranes, tubular cell necrosis and apoptosis [51]. The total score is the average of all tubular scores. A total of 200 tubuli per section were evaluated.

### Immunohistochemical staining

To perform immunohistochemical staining, rabbit anti-hypoxia inducible factor-1α (HIF-1α) polyclonal antibodies, rabbit anti-CD34 polyclonal antibodies (1∶50) (Boster Biotechnology), rabbit anti-TGF-β1 polyclonal antibodies (1∶50) (Santa Cruz Biotechnology), rabbit anti–MCP-1 (1∶100, Boster Biotechnology), rabbit anti-E-cadherin protein (1∶100, Zhongshan, China), rabbit anti-Collagen-I (1∶100, KeyGEN Bio TECH, China), and rabbit anti–Ki67 (1∶50, Boster Biotechnology) were used for immunohistochemical procedures. All the above staining for immunohistochemical were performed using the PV6001 and DAB kits (Zhongshan, China) according to manufacturer's instructions. Hematoxylin was used for counterstaining. Ten discontinuous visual fields of the outer cortex of the kidney were also randomly selected under the microscope for each section. The integrated optical density (IOD) total of each visual field was determined using the Image 6 Pro Plus System (Media Cybernetics, USA) [52]. Besides, goat anti-VEGF monoclonal antibodies (1∶100) (Boster Biotechnology) and rabbit anti-WT1 monoclonal antibodies (1∶100) (Boster Biotechnology) were used as primary antibody for double immunostaining, followed by which were FITC-AffiniPure rabbit Anti-goat IgG (H+L) (Jackson) and Rhodamine (TRITC)-conjugated AffiniPure goat Anti-rabbit IgG (H+L) (Jackson) used as secondary antibody. Before mounting these sections, tissues were counterstained with DAPI. Ten discontinuous visual fields of the outer cortex of the kidney were randomly selected under the fluorescence microscope for each section. The integrated optical density (IOD) total of each visual field was determined using the Image 6 Pro Plus System (Media Cybernetics, USA) as described above.

To investigate the capillary density, tissue samples stained for CD34 expression were examined using light microscopy at 40× magnification according to methods described previously [19]. PTCs that stained positive for CD34, an endothelial cell-specific marker, were counted in five chosen microscopic fields on each slide; capillary density was presented as the average number of capillaries/0.065 mm^2^.

### Western blot analysis

Renal samples were lysed in RIPA (Bytotime, China) on ice and then centrifuged at 10,000 rpm for 15 min at 4°C. The protein samples (100 µg protein/lane) were electrophoresed through 7% polyacrylamide gels and then transferred to nitrocellulose membranes (Amersham SA, France). The membranes were blocked in 5% skim milk powder for 1 h, and then incubated overnight at 4°C with primary monoclonal mouse anti-VEGF antibodies (1∶100), rabbit anti-HIF-1α antibodies (1∶100),rabbit anti-TGFβ1 antibodies (1∶100),rabbit anti–MCP-1 (1∶100), rabbit anti-E-cadherin protein (1∶100),rabbit anti-Collagen-I (1∶100). The immune complexes were detected using alkaline phosphatase-conjugated secondary antibodies (Zhongshan, China) and a BCIP/NBT Alkaline Phosphatase Color Development Kit (Beyotime, China). Positive immunoreactive bands were quantified densitometrically and normalized using beta tubulin.

### Proliferation and Apoptosis Measurements

The number of proliferating cells at each time point was detected using PCNA and Ki67 Staining Kits (Invitrogen). All staining procedures were performed according to the manufacturer's instructions. Briefly, after deparaffinization, rehydration and blocking, the slides were incubated with biotinylated rabbit anti-PCNA primary antibodies for 1 hour and rabbit anti-Ki67 primary antibody for 2 hours at room temperature; slides were then incubated with Streptavidin-HRP and developed using DAB substrate. In each of the experimental groups, the proliferative nuclei were counted as a fraction of the total number of nuclei present in the section using consecutive, non-overlapping fields of PCNA-stained specimens and Ki67-stained specimens.

The number of apoptotic cells was determined using TUNEL Apoptosis Detection Kits for paraffin-embedded tissue sections; all staining procedures were performed according to manufacturer's instructions. All of the reagents were furnished by the kit. Briefly, after deparaffinization and rehydration, the slides were incubated with Proteinase K solution for 30 min at 37°C, washed in PBS for 2 min, and then blocked. Slides were next incubated with a TUNEL reaction mixture for 1 hour at 37°C, followed by the Streptavidin-HRP solution for 30 min at 37°C. Last, slides were developed with the DAB substrate. In each of the experimental groups, the apoptotic nuclei were counted as a fraction of the total number of nuclei present in the section using consecutive, non-overlapping fields of TUNEL-stained specimens.

### Statistical analysis

All data are displayed as the mean values with their standard deviation indicated (mean±SD). The statistical analysis for the determination of differences in the measured properties between groups was accomplished using one-way analysis of variance (ANOVA) followed by a Dunnett's post hoc test; differences were considered to be statistically significant when the p-values were less than 0.05 (p<0.05). All statistical analyses were performed using SPSS 16.0.
